# Patients’ and practice nurses’ perceptions of depression in patients with type 2 diabetes and/or coronary heart disease screened for subthreshold depression

**DOI:** 10.1186/s12875-018-0870-y

**Published:** 2018-12-23

**Authors:** Alide D. Pols, Karen Schipper, Debbie Overkamp, Harm W. J. van Marwijk, Maurits W. van Tulder, Marcel C. Adriaanse

**Affiliations:** 1 Department of Health Sciences, Amsterdam Public Health Research Institute, Faculty of Science, Vrije Universiteit Amsterdam, Amsterdam, the Netherlands; 2 Amsterdam Public Health Research Institute, Department of General Practice & Elderly Care Medicine, Amsterdam UMC, Vrije Universiteit Amsterdam, Amsterdam, the Netherlands; 3 Amsterdam Public Health Research Institute, Department of Medical Humanities, Amsterdam UMC, Vrije Universiteit Amsterdam, Amsterdam, the Netherlands; 4 Division of Primary Care and Public Health, Brighton and Sussex Medical School, Mayfield House, University of Brighton, Brighton, UK

**Keywords:** Qualitative study, Depression, Type 2 diabetes mellitus, Coronary heart disease, Illness perceptions, Need for care

## Abstract

**Background:**

Comorbid depression is common in patients with type 2 diabetes (DM2) and/or coronary heart disease (CHD) and is associated with poor quality of life and adverse health outcomes. However, little is known about patients’ and practice nurses’ (PNs) perceptions of depression. Tailoring care to these perceptions may affect depression detection and patient engagement with treatment and prevention programs. This study aimed to explore patients’ and PNs’ perceptions of depression in patients with DM2/CHD screened for subthreshold depression.

**Methods:**

A qualitative study was conducted as part of a Dutch stepped-care prevention project. Using a purposive sampling strategy, data were collected through semi-structured interviews with 15 patients and 9 PNs. After consent, all interviews were recorded, transcribed verbatim and analyzed independently by two researchers with Atlas.ti.5.7.1 software. The patient and PN datasets were inspected for commonalities using a constant comparative method, from which a final thematic framework was generated.

**Results:**

Main themes were: illness perception, need for care and causes of depression. Patients generally considered themselves at least mildly depressed, but perceived severity levels were not always congruent with Patient Health Questionnaire 9 scores at inclusion. Initially recognizing or naming their mental state as a (subthreshold) depression was difficult for some. Having trouble sleeping was frequently experienced as the most burdensome symptom. Most experienced a need for care; psycho-educational advice and talking therapy were preferred. Perceived symptom severity corresponded with perceived need for care, but did not necessarily match help-seeking behaviour. Main named barriers to help-seeking were experienced stigma and lack of awareness of depression and mental health care possibilities. PNs frequently perceived patients as not depressed and with minimal need for specific care except for attention. Participants pointed to a mix of causes of depression, most related to negative life events and circumstances and perceived indirect links with DM2/CHD.

**Conclusion:**

Data of the interviewed patients and PNs suggest that they have different perceptions about (subthreshold) depressive illness and the need for care, although views on its causes seem to overlap more.

## Background

Comorbid depression in patients with type 2 diabetes (DM2) and/ or coronary heart disease (CHD) is a major health issue. The risk of depression in these patients is approximately double compared to the general population [1, 2]. This comorbidity is associated with diminished self-care and medication adherence [3, 4], poorer quality of life [5], and increased mortality [6, 7]. Similar negative effects are seen with comorbid subthreshold depression [8], defined as clinically relevant depressive symptoms without fulfilling the criteria for major depressive disorder (MDD). Subthreshold depression is present in approximately one third of the patients with DM2 and/or CHD [9–11] and is the strongest predictor for the onset of MDD [12, 13].

Despite its negative impact, depression often remains under-recognized, under-discussed and undertreated in the general population [14]. The detection of comorbid depression in patients with long term physical conditions, like DM2 and CHD, is even more challenging as symptoms can overlap [15, 16]. Therefore, in clinical guidelines, various organizations have suggested screening for depression to improve detection rates [15, 17, 18]. However, at present, there is no substantial evidence that this approach is effective [19, 20]. Reducing the burden of depression by preventing the influx of new cases is a promising strategy, particularly through early recognition and treatment of patients at risk (indicated prevention), such as those with subthreshold depression. Meta-analyses have shown that preventative psychological interventions can overall reduce the incidence of MDD in comparison to control groups [12, 21].

Offering preventative psychological interventions in a stepped-care format could be an efficient approach, which also fits well with current task shifting and delegating trends. In primary care in the Netherlands, most GPs work with psychological practice nurses (those who provide low-intensity mental health care) and somatic practice nurses (those who largely focus on general physical care), who are generally located in the same building. This internationally unique integrated primary care team aims to provide local community-based continuity of care [22, 23]. In stepped-care, patients start with minimally intensive evidence-based treatments and progress is monitored systematically. Those who do not improve adequately, step up to a treatment of higher intensity [24]. Many guidelines endorse this stepped-care principle for depression treatment [15, 25, 26], but the evidence on the effectiveness of prevention is limited and conflicting. While effective in reducing the incidence of MDD in elderly or visually impaired populations [27–29], it was not superior to usual care in other elderly, diabetic or primary care populations [9, 30–32]. Recently, we conducted a randomized controlled trial in which we evaluated whether a pragmatic, nurse-led stepped-care program was effective in reducing the incidence of MDD at 12-months of follow-up in comparison with usual care among patients with DM2 and/or CHD and subthreshold depression (Step-Dep study) [33]. The stepped-care approach was not superior to usual care after one year [34]. Consecutively, qualitative research was conducted to gain a deeper understanding of these results. A process evaluation was conducted in which we explored both patients’ and practice nurses’ experiences with the Step-Dep program using the RE-AIM model which assesses five dimensions of an intervention: reach, efficacy, adoption, implementation, and maintenance [35]. We focused on barriers and facilitators of the implementation of the Step-Dep program [36] next to a more conceptual exploration of how DM2/CHD patients and practice nurses perceived comorbid depression.

More insight into patients’ perceptions of depression in long-term conditions is of great value. Recent systematic reviews have suggested that the limited understanding we currently have contributes to many of the encountered difficulties in depression care [16, 37]. Differences between patients’ and health care providers’ perceptions further add to these difficulties [38]. As most patients with depression and DM2 and/or CHD are managed in primary care in the Netherlands, knowledge of these patients’ and their health care providers’ perceptions of this comorbidity is important, but only a few studies investigating this have been conducted [16, 39–44]. The most used theoretical framework in such studies on perceptions or illness representations is the Common Sense Self-Regulation Model of Health and Illness by Leventhal et al. [45]. This framework states that patients construct their own perceptions of the causes and consequences of the illness, its time-course, the feasibility of controlling or curing it, how it affects one’s identity and emotions, and how well the illness is understood. This helps patients to make sense of their illnesses and serves as the basis for coping. Previous studies have mainly focused either on the patient perspective and their experienced relationship between these disorders (the ‘cause’ item of the model) [16, 39–42], or on health care providers’ views of managing depression in these long-term conditions (‘cure-control’) [43, 44]. Studies exploring aspects like illness perceptions (‘identity’) and perceived need for care (‘cure-control’), or comparing patients’ and health care providers’ perceptions are lacking. Yet, to improve patient engagement in future indicated prevention programs, knowledge on whether targeted patients perceive themselves as ‘ill’ and if they perceive a need for care, seems crucial. Moreover, better understanding of both caregivers’ and patients’ perspectives, as well as the differences between them, may enable prevention programs to be more tailored to these perceptions, potentially improving depression care [46]. Therefore, this study aimed to investigate patients’ and practice nurses’ perceptions of depression in patients with DM2 and/or CHD screened for subthreshold depression.

## Methods

### Step-dep study

This qualitative study was part of the Step-Dep study, which consisted of both a pragmatic cluster randomized controlled trial with economic evaluation, whose design [33], results [34] and process evaluation [36] have been described elsewhere. The qualitative process evaluation consisted of semi-structured face-to-face interviews with 24 participants of the intervention arm. As an extension to the ‘reach’ dimension of the RE-AIM model [35], which describes study participants’ characteristics and compares them to the target population, patients‘ and practice nurses’ perceptions of depression were thoroughly explored and reported in this article.

### Participants and recruitment

We interviewed all the practice nurses involved in the implementation of the Step-Dep intervention. Amongst them were both psychological practice nurses and somatic practice nurses. Psychological practice nurses provide low-intensity mental health care for primary care patients. Somatic practice nurses provide chronic disease management in the GP practice of patients with physical long-term conditions like DM2, CHD, chronic obstructive pulmonary disease (COPD) and asthma. In the Netherlands, the educational programs for these two types of practice nurses are separate and generally take one year after an appropriate pre-registration education of four years at a University of Applied Sciences.

All Step-Dep study participants had a diagnosis of DM2 and/or CHD, hence the term ‘patient’ used in this paper. In addition, these patients screened positive on subthreshold or mild depression, which was defined as a Patient Health Questionnaire 9 (PHQ-9; range 0–27) score of six or more [47, 48] without evidence of a major depressive disorder according to the Mini International Neuropsychiatric Interview (MINI) [49, 50]. We used purposive sampling [51] to recruit a diverse sample of patients in order to elicit as many different views as possible on the pre-specified topics of the Common Sense Self-Regulation Model of Health and Illness model [45]. Based on a literature review of factors influencing depression incidence and outcome, we selected patients on: gender, age, presence of DM2 and/or CHD, self-reported history of depression, self-reported current depression, level of education, baseline depression severity (PHQ-9), baseline anxiety severity (HADS-a), baseline quality of life score (EQ5D), baseline social support scores, locus of control scores. In addition, we selected patients from different urban and rural residential areas.

Both patients and practice nurses were asked by an investigator (AP or DO) to participate in the interviews by phone. All initially selected participants agreed to be interviewed, except for three patients, who were either suffering from a terminal illness or had a terminally ill partner. Three other patients were then asked and all agreed to participate.

### Data collection

The interview topic guide (Appendix 1) was both based on the study aims of the process evaluation [36], and included the assessment of patients’ and practice nurses’ perceptions of (subthreshold) depression in DM2/CHD. For the latter, open-ended questions were formulated (Appendices 2 and 3) that drew upon the Common Sense Self-Regulation Model of Health and Illness [45]. We focused on illness perception (‘identity’), need for care (‘control-cure’) and causes of depression and the interplay with their DM2/CHD (‘cause’). These topics were considered to be most clinically relevant by the research-team, because of both patients’ and practice nurses’ interim feedback during the Step-Dep study, and the research questions that remained after its effectiveness analyses [34].

Two researchers (AP and DO) conducted all the interviews from September to November 2015. After consent, all interviews were anonymized, digitally recorded, transcribed verbatim and entered into Atlas.ti 5.7.1 for analysis and data management. Interviews took place at venues preferred by participants; at home (patients *n* = 11), at the GP practice (practice nurses *n* = 8) or at the VU University Medical Center in Amsterdam (patients *n* = 4, practice nurses *n* = 1) and lasted about 45 min each. AP kept at a reflective journal to be of aid in later analyses. A member check was performed and all participants but one (a patient who could not be reached despite multiple attempts) confirmed the content of the summary sent by mail to be representative of the interview [51]. Data saturation was reached after interviewing 11 patients. Four more patients were subsequently interviewed to confirm this [51]. All nine participating practice nurses in the intervention arm were interviewed, with data saturation reached after the eighth interview.

### Data analysis

The process of data collection and analysis was iterative, as data analysis was concurrent with data collection to enable the incorporation and validation of relevant emerging themes into subsequent interviews. Elements from a responsive evaluation were used. This approach provides the opportunity to explore the multiple perspectives of involved stakeholders and to create a rich and multi-layered understanding of a phenomenon [52]. An important notion in responsive evaluations is that stakeholders are involved in the study and that the perspective of patients is taken into account [52]. Along the research process, data were subject to a inductive thematic analysis [53, 54] .

First, codes were attached to citations related to specific (sub)topics (open coding), leading to a set of descriptive topics per transcript. Then, all codes of all transcripts were compared and redefined, and clustered into themes and subthemes (axial coding) and, overarching themes were formulated (selective coding). Next, similarities and differences between cases were identified (cross case analysis of constant comparison [55]). Patient and practice nurse transcripts were analyzed separately, but comparisons were made across data sets. Two researchers (AP and DO) analyzed the data individually, and relevant themes were agreed upon.

## Results

### Participants

Table 1 shows the patient and practice nurse characteristics as measured at baseline of the Step-Dep study. Of the 15 participating patients, eight were female. The average age was 62, ranging from 48 to 84 years. PHQ-9 scores at inclusion varied from seven to 16 and were 10.9 on average. 11 patients reported a history of depression and five patients a current depression. Of the nine practice nurses interviewed, six were psychological practice nurses, three were somatic practice nurses and one of the latter had been a psychological practice nurse before. The average number of treated Step-Dep patients per practice nurse was 11 and varied from 3 to 24. Additional data can be found in Table 1.

**Table 1:** Patient (*n* = 15) and practice nurse (*n* = 9) characteristics at inclusion Step-Dep study

Patients		
Gender (n)	Female	8
	Male	7
Age	Range	48–84
	Mean	62
Chronic disease (n)	DM2	9
	CHD	10
	DM2 and CHD	4
Number of long-term conditions	Range	1–9
	Mean	3
Level of education (n)	Low	4
	Average	5
	High	6
History of depression (n)	Yes	11
	No	4
Self-reported depression (n)	Yes	5
	No	10
Depression severity PHQ-9 at inclusion	Range	7–16
	Mean	10,9
Anxiety HADS-A	Range	2–15
	Mean	8
Quality of life EQ5D	Range	0,39-0,92
	Mean	0,72
Social support	Range	34–55
	Mean	45
Locus of control	Range	5–21
	Mean	14
Practice nurses		
Gender (n)	female	7
Type (n)	Psychological practice nurse	6
	Somatic practice nurse	3
Number of patients treated during Step-Dep	Range	3–24
	Mean	11
Years of relevant professional experience as health-care provider	Range	3–30
	Mean	16,3

Abbreviations: *CHD* = Coronary Heart Disease, *DM2* = Type 2 Diabetes Mellitus, *PHQ-9* = Patients Health Questionnaire 9 score (range 0–27, higher scores indicating more severe depression), *HADS-A* = Hospital Anxiety and Depression Scale (range 0–21, with higher scores indicating more severe anxiety), *EQ5D* = EuroQol-5D (range 0–1, with higher scores indicating higher quality of life), social support (range 0–48, higher scores indicating more perceived social support), locus of control (range 0–20, higher scores indicating a more external locus of control)

### Main themes

The results of this study are presented by three main themes: 1) illness perception, 2) need for care and 3) causes of depressive symptoms. As the focus of this study was on perceptions of mental health, this is implied in both the concept of (mental) illness perception and need for (mental health) care. An overview of the main findings per theme and the corresponding interview questions can be found in Table 2. For each theme, the most illustrative quotes were selected. Per quote, the main interviewee characteristics are described; P is used for patients and N for practice nurses. Full (anonymized) interviewee details can be found in Appendix 4.

**Table 2:** Overview of themes, questions and results

Themes	Questions	Results
Illness perception (identity**)**	Patient • How would you describe your mental state before starting Step-Dep? • If not depressed: please tell more about it? • If depressed: please tell more about it? Did it influence your life? PN • How did you view their mental state/ depressive symptoms? • Did patients recognize themselves in the depressed profile?	• Patients’ and PNs’ perceptions of depressive symptom severity varied from not to severely depressed and were not always congruent with PHQ-9 scores at inclusion • Almost all patients considered themselves at least mildly to moderately depressed • PNs frequently perceived their patients as ‘not depressed’ • Patients sometimes needed time to talk about and reflect on their mood • Work experience perhaps influenced PNs’ perceptions of patients’ depressive symptoms • Many patients did not initially realize that the mental state they were in was a level of depression • Patients preferred using their own words to describe their mental state, some terms were not connected to mood. • Sleeping was frequently pointed out as the most burdensome symptom
Need for care (cure/control)	Patient • Were you in need of care/ a preventive program to improve depressive symptoms? • How would it have been, if you had not received an invitation for Step-Dep? • What were your expectations/ hopes from the program? • What would your care of choice have been like? And to improve depressive symptoms? PN • Were the patients in need for care for depression? Other need for care? Why? Why not?	• Most interviewed patients experienced a need for care and preferred psycho-educational advice and talking therapy • PNs frequently said that patients had minimal need for specific care and mostly needed attention • In patients, perceived symptom severity corresponded with perceived need for care, but did not necessarily match help-seeking behaviour • Barriers to seek care: ○ Not realizing that mental state is a level of depression ○ Experienced stigma of depression ○ Unfamiliarity with mental health care ○ Experienced barriers discussing mental problems with GP
Depression causes (cause)	Patient • Is there a relationship with your chronic disease? How? • What do you think caused your depressive symptoms? • How is your mental state now? If improved: what are the reasons for that? PN • How do you view the relationship with the chronic disease? What coping strategies do patients have with a chronic disease? • What are causes of depressive symptoms? • If the depressive symptoms improved in your patients; what was the reason?	• Most patients and PNs appointed a mix of causes of depression • Most were related to negative life events and circumstances • Many PNs and patients perceived indirect links with long-term conditions via: ○ physical limitation ○ changed future perspectives ○ difficulties with acceptance of diagnosis of a long-term condition

#### Illness perception

In general, patients and practice nurses perceived patients’ depressive symptom severity prior to the start of Step-Dep as varying widely, ranging from ‘not depressed’ to ‘severely depressed’. Patients’ perceptions of their symptom severity did not necessarily correspond with their individual PHQ-9 scores at inclusion. It was more common for the PHQ-9 scores to be higher than the perceived symptom severity than the other way around, but both occurred. When asked whether patients recognized themselves in the ‘subthreshold depression’ profile they were screened on, three patients responded at first that they had not felt depressed at all. One of them explained that she only screened ‘positive’ (with a relatively high score of 11) on the PHQ-9 by scoring on physical symptoms, unrelated to her mood, caused by her multiple chronic diseases. However, during the interviews, the other two patients eventually explained that they had been somewhat down or ‘sombre’. In Dutch primary care depression guidelines and patient information, ‘sombre’ is the most frequently used term to describe all severity levels of depression [56]. It seemed that these two patients needed time to feel comfortable enough to open up and reflect on their mood, which could be problematic in short consultations in primary care.

***“*** *It was not as if I was in a sombre mood when I decided to participate. […] Well yes, that was when I was not feeling too happy…” (P2, female, CHD)*

All the other interviewed patients did experience some level of depression and indicated that this had a significant impact on their daily lives. About half the patients thought their mood matched the subthreshold depressed profile well and confirmed that they had felt mildly to moderately depressed. Yet many others described themselves as fully depressed.

*“I was feeling really miserable. Too often feeling sombre and too tired. A complete lack of energy, just a wreck. I had trouble sleeping and concentrating. I was just not happy. Not a fun person anymore, in my opinion. (laughs). There was no room for anything else. I think I was actually barely hanging on. Yes, I was certainly depressed.” (P5, female, DM2)*

Whereas almost all patients would have labelled themselves as at least mildly to moderately depressed, practice nurses frequently perceived their patients as ‘not depressed’. Two very experienced psychological practice nurses, who treated more Step-Dep patients than other practice nurses, even reported that virtually none of their patients were depressed.

*“But I did not consider them depressed. That is something you can sense, or taste almost. No.” (N7, psychological PN)*

One of these practice nurses questioned whether her perception of her patients’ depressive symptoms was influenced by her working experience: *“As I am used to working with some more severe problems, I thought: ‘Am I missing something here?’” (N2, psychological PN).* In contrast, a few practice nurses called their patients chronically (mildly) depressed and one somatic practice nurse thought all her patients had severe depressive symptoms.

In terms of acknowledging, labelling and naming symptoms as part of ‘depression’, patients initially had difficulty realizing that their mental state was actually a level of depression. Filling out the PHQ-9 questionnaire as part of the screening process of Step-Dep and reflecting on that, seemed to help patients to identify their negative mental state as a (subthreshold) depression.

*“Looking back, I wouldn’t have thought that I was that… how should I phrase that…sombre. That actually shocked me at times. To realize that I seemed quite negative. And I actually was negative back then.” (P9, male, DM2 & CHD)*

The following extract illustrates how some practice nurses experienced this in their patients as well.

*“Due to that questionnaire, they would say: ‘My gosh, all this time, I have been depressed without knowing.’ The best example was this one patient who had a massive score and was like: ‘My goodness, what is the matter with me?’ Well, she had been feeling miserable, but had not connected the dots.” (N3, psychological PN)*

Many patients however, who did recognize their mental state as a mild to moderate depression, rejected the term ‘sombre’ to describe it. It felt like a stigma for some or just too exaggerated for others. The following quote illustrates this.

*“Sombre would be exaggerating, but I sure wasn’t cheerful. Not a happy lad and at the same time seeing a psychologist.” (P11, male, DM2)*

Almost all patients would spontaneously use other words to describe their low mood, even the patients that labelled themselves as ‘fully depressed’.

*“took a bad turn” (P10, male, DM2) “rough times” (P11, male, DM2) “continuous sorrow” (P13, male, CHD) “down and out” (P12, female, DM2 & CHD) “wrecked” (P12, female, DM2 & CHD)*

Some of the terms they used, were not even necessarily connected to a depressed mood.

*“loss of self-confidence” “stress” (P11, male, DM2) “ burdensome worries” (P1, female, CHD) “burn-out” (P12, female, DM2 & CHD)*

A striking number of patients reported troubled sleeping and described this as their most burdensome symptom. The following quotation illustrates a perceived link between trouble sleeping and depression.

*“Trouble sleeping. You fall into a downwards spiral, you get so tired, chronically tired I would say. It makes it so easy to stay underneath the covers in the morning, drifting off to depression.” (P15, female, CHD)*

#### Need for care

Most interviewed patients indicated that they experienced a need for (mental health) care prior to the start of the Step-Dep study. In general, the perceived symptom severity matched the level of perceived need for care. Many practice nurses explained that the need for care varied considerably between patients, and usually corresponded with their perception of the patients’ symptom severity. However, the general opinion amongst practice nurses was that the majority of patients had minimal need for specific care and mostly needed attention.

*“The majority did not have a need for care, no. And those who did, were so depressed that they needed clinical treatment.” (N7, psychological PN)*

The majority of patients cited practical advice and someone to talk to as their preferred modes of care. Practical advice entailed ways to improve their mood, for example through physical exercise and activity planning, and often concerned handling sleeping problems.

*“Just talking to someone, every other week, for half an hour or an hour. To get some practical advice of (name practice nurse) on how to cope with trouble sleeping for example. For her to say: ‘Why don’t you try this’, that really works.” (P15, female, CHD)*

Many patients did not have a clear idea who the person ‘to talk to’ would be, but psychotherapists were most frequently mentioned.

*“I did not know much about it, except for the term ‘psychotherapy’.” (P7, male, DM2)*

Whereas patients mainly emphasized their need for practical psycho-educational advice and talking therapy, practice nurses reported that patients predominantly needed attention.

*“I often reckoned that maybe they just needed some attention. Not to be negative or anything. Just to have somewhere and someone to talk to without sparing that someone, like they would have to with a partner or family member. The freedom to just talk. A need for attention.” (N2, psychological PN)*

Such mismatches in patients’ and practice nurses’ views on how much and which care is needed potentially jeopardizes patient engagement in offered care.

An interesting finding from the patients’ interviews was that the perceived symptom severity and the corresponding perceived need for care did not necessarily match patients’ own predictions of or actual help-seeking behaviour. While most patients experienced a need for care, many did not and would not have asked for it. Patients explained that they experienced barriers that withheld them from seeking care. These appointed barriers were often also perceived by practice nurses. Many of the barriers indicated dealt with the taboo and social stigma of depression. The following quotation illustrate a variety of these.

*“Being a true ‘Twent’ (Dutch word for someone from the eastern province of the Netherlands) I never reveal what I am truly feeling.” (P10, male, DM2)*

*“I never would have asked for that kind of help myself. Growing up, I was taught not to complain. Especially not about mental problems, because that is just all in your head and therefor something you should resolve on your own. […] To overcome the idea of ‘You used to be normal, yet now you have become a psychiatric patient’ […] The stigma already completely surrounds you.” (P7, male, DM2)*

*“So many of them were of a certain age, when society used to say ‘Take it like a man, stop complaining.’ And so many would lead their lives according to these social codes, bearing their problems in silence.” (N1, psychological PN)*

In addition, as patients would often not realize that they were depressed, they were unaware that they could ask for help.

*“I wonder if I would have looked for any help, since I was just so used to feeling like that. I just feel so much better now. It makes me think: ‘Darn, things were definitely not alright back then.’ But, it was normal for me.” (P5, female, DM2)*

*“But in the end, there were quite a few who did have a need for care. But apparently, they had not acted upon it yet. It had not reached their frontal lobe yet, so to say. Not up to the point where they would say: ‘I need to do something about this, I should make an appointment.’”(N3, psychological PN)*

Patients also mentioned barriers that practice nurses did not. Some patients explained that they were unfamiliar with mental health care and its possibilities for help. Other patients mentioned difficulties talking to their GPs about mental problems. Patients felt that GPs mainly focused on physical disease, or they experienced a lack of time and space, or a lack of continuity of care to discuss mental issues with their GP.

*“I guess because I was unfamiliar with that area of health care, I would not have looked for it.” (P9, male, DM2 & CHD)*

*“In my experience, GP’s are always short on time. That makes it really difficult to discuss that kind of problems. Because GPs, like mine, are so busy already and work part-time too, that you always see a different one, which I find very disturbing.” (P1, female, CHD)*

#### Causes of depressive symptoms

Both patients and practice nurses indicated various causes of depressive symptoms, both related and unrelated to long-term conditions, and most said that a mix of these leads to depression. The most frequently mentioned causes were serious life-events like divorce, bereavement or childhood traumas, as well as negative circumstances like job loss or work pressure. Other, less frequently named causes were personal traits like personality, genes or character, aging and loneliness.

*“Those life events obviously had an impact on their quality of life and appealed to their coping mechanisms.” (N1, psychological PN)*

*“It was caused by job insecurity, financial problems or by thyroid medication that needed adjusting. They would appoint very specific problems and say: ‘The way I felt, was a reaction to those problems.’ Circumstances, yes.” (N2, psychological PN)*

Many patients and practice nurses experienced indirect links between long-term conditions and depression. Physical limitations caused by DM2, CHD or other chronic diseases, along with their impact on daily life, were seen as the most prominent indirect causes of depression. Interviewees did not necessarily presume a ‘linear’ relation between the severity of these limitations and depressive symptoms. Changed prospects of the future due to a chronic disease formed another important indirect cause. Further, both patients and practice nurses explained how ‘mourning’ the diagnosis of a chronic illness could lead to depression, in which acceptance problems played a dominant role.

*“I used to walk 20 to 25 km with a friend every other week. That used to be so easy for me, but I can’t anymore. The fact that we had to turn around, that I couldn’t finish that specific walk and had to take a short-cut back… That had a considerable impact. It did not cheer me up at all, to the contrary.” (P6, male, CHD)*

*“But even in those people with severe limitations, it would not necessarily have that much of an impact. I am remembering this lady who was severely limited, but was so incredibly active. (laughs) In her case, it did not influence her mood, per se.” (N4, psychological PN)*

*“I don’t really feel those glucose levels. I know the diabetes is there and I realize its consequences, which is possibly the most frightening aspect for me. People say that it is a secret assassin, and that is true, actually.” (P10, male, DM2)*

*“It is a kind of ‘mourning’ process that you have to go through, to reach a state of acceptance of your losses, like your energy levels, at work, things you used to be able to do. You have to learn to accept that you won’t be able to do all of that anymore. Well, that was my biggest problem.” (P7, male, DM2)*

Very few patients and practice nurses directly linked DM2 and/ or CHD to depression.

*“That (her and her husband’s chronic diseases) absolutely has it effect on the things you want to do or the way you feel. I do believe that.” (P12, female, DM2 & CHD)*

*“Well, I have seen how being chronically ill just leads to a depressed mood.” (N8, somatic PN)*

There were also some patients and practice nurses who believed that depression is not related to DM2 or CHD.

*“Well, it didn’t even cross my mind, that is how important it is to me. I have a hint of diabetes. (laughs) I just use one pill a day. For me, it is such a none-issue, that it hadn’t even occurred to me.” (P11, male, DM2)*

Only one practice nurse reckoned that the diagnosis of a long-term condition itself could have an anti-depressant effect.

*“I did not see that presumed relation, or hardly. It is very well possible that people adjust their lifestyle, and realize the impermanence of life…that it is a wake-up call and acts as an anti-depressant.” (N7, psychological PN)*

## Discussion

This qualitative study explored patients’ and practice nurses’ perceptions of the construct of ‘depression’ in patients with DM2 and/or CHD screened for subthreshold depression. Our overall analysis is that better understanding of how chronically ill patients make sense of depressive symptoms or illnesses, in view of their need for care and in view of how they see the symptoms in the context of their lives (the ‘causes’) is crucial for the implementation of mental health care into chronic disease care. Perhaps practice nurses can also be better trained for this.

### Illness perception

In general, the interviewed patients considered themselves at least mildly depressed, whereas practice nurses, interestingly, frequently perceived patients as not depressed. This discrepancy is perhaps partially caused by the fact that psychological practice nurses are used to working with patients with quite severe depression and a clear request for help. Step-Dep patients, on the other hand, were pro-actively selected on the presence of subthreshold depression on a self-report questionnaire. Furthermore, previous research suggests that somatic practice nurses sometimes experience a lack of competence to adequately recognize and handle mental problems in chronically ill patients [36]. However, this could also be part of a more widespread phenomenon, as it is has been described before that many caregivers have the tendency to ‘normalize’ depression in patients with long-term conditions [43, 44]. In addition, some patients initially did not recognize their mental state as a level of depression, which might prohibit them from disclosing their depressive symptoms to caregivers. This has been observed in other studies as well [37, 39]. These studies suggest that patients might ‘refuse’ to recognize and acknowledge their depression due to an inner conflict of their ideal self-identity and perceiving themselves as a person with depression (ego dystonia in Freudian terms). In this study, we have also observed the opposite as in some patients sombre feelings would be present for so long, that they accepted these as normal (egosyntonic) and therefore failed to recognize these as a level of depression.

Perceived depressive symptom severity was not always congruent with PHQ-9 scores at inclusion. Both over- and underestimation by the PHQ-9 of depression severity was perceived. Even though the PHQ-9 is a validated instrument to screen for mild depression in the chronically ill using a cut-off of 6 [47, 48], our findings could indicate that, in these specific long-term conditions, the discriminative properties of this method were not optimal. A recent study in a population of patients with DM2/CHD, found optimal cut-off scores for minor and major depression to be within a small range of 8 and 10 respectively [57]. This suggests that the PHQ-9 might not be specific enough to distinguish minor from major depression for scores in this range. A higher cut-off score of 8 might be necessary in order not to over-diagnose mild depression in patients with DM2/CHD, as symptom of the somatic diseases and depression, like fatigue and altered appetite, can overlap. Also, there is an association between depressive symptoms and distress related to long-term conditions [58], such as diabetes distress [59, 60]. A complex finding of our study was that even though patients explained they felt mildly to moderately depressed, they independently labelled their mental state differently than ‘depression’. It seems likely that patients actually do suffer from depressive symptoms, but prefer using different labels like ‘stress’ or ‘sleeping disorders’, as they perceive these as less stigmatizing than ‘depression’. However, since the specificity of the PHQ-9 with a cut-off of 6 was found to be only 55% [57], our findings raise questions over whether it discriminates enough between mild depression and mild forms of other psychological problems, like anxiety, burn-out or sleeping disorders.

In this study, many patients expressed both the heavy burden of sleeping problems and the wish to alleviate it. Problems with sleeping are classic symptoms of depression, but the associations between disturbed sleep and depression [61] or long-term conditions [62], like CHD and DM2, have also been well established. While the underlying mechanisms of the relationships between these conditions and their implications for rational therapeutics should be further explored [63], addressing sleeping problems seems a promising starting point for the delivery of mental health care for most patients with depressive symptoms.

### Need for care

Perceived need for care coincided with perceived symptom severity, but often did not match help-seeking behaviour. Although most patients experienced a need for care, preferring psycho-educational advice and talking therapy, many would not have sought such care if it had not been offered pro-actively. Patients blamed several experienced barriers. The perceived stigma of depression was the most important barrier, but the initial lack of awareness about depression and mental health care options, and perceived difficulties to discuss mental health issues with GPs, were also mentioned. In previous studies, experienced stigma and taboo of depression were found to form important barriers to both help-seeking and disclosure of depressive symptoms [16, 37, 39, 43]. Whereas the appointed barriers apparently withheld patients from actively seeking care, it did not seem to withhold them from accepting care by participating in the Step-Dep program. Pro-actively offering care therefore appears to be an appropriate approach to overcome such barriers. However, we cannot exclude the possibility that the motivation to contribute to research was actually pivotal in their decision to participate in the program. The process evaluation of Step-Dep revealed that all patients appointed the contribution to research as (one of the) primary motivators to participate. Only less than half named the need to improve their mood as a primary motivation [36]. Yet, given the importance and magnitude of perceived stigma of depression, it seems more likely that naming the contribution to research instead of experienced depression as the main motivator felt less stigmatizing for some and the pro-active offer of care facilitated the acceptance of care.

### Causes of depression

The interviewees in this study cited a mix of causes leading to depression. The perceived importance of the contribution of negative life events and circumstances to the development of depression is in line with findings from the review by Anderson et al. [37]. A direct causal link between long-term conditions and depression was largely not supported in our study. This is in contrast with the views of the elderly interviewed by Bogner et al. [42], who perceived that their long-term condition directly caused depression and vice versa. Patients in this and several other studies reported that long-term conditions can lead to depression, not the other way around. In these patients’ views, long-term conditions caused depression indirectly, via the burden of physical limitations [16, 41], diminished future perspectives [16] and difficulties accepting the long-term condition diagnosis. The latter was frequently explained in our study as part of the ‘mourning’ process. Both patients and caregivers frequently referred to terms like ‘mourning’ and ‘acceptance’ when describing the response to chronic illness [64], which are originally derived from the Kubler-Ross’ grief model [65]. These outcomes, however, are not supported by multiple studies showing that the diagnosis of DM2 by screening does not have significant psychological impact [66, 67]. Still, tuning into patients’ perceptions of the causes might facilitate the conversation on depression. In chronic disease care, starting points could therefore be the impact of the diagnosis, physical limitations, or the impairment of future perspectives. However, patients who do not perceive any link between their long-term condition and depression might not disclose depressive symptoms in integrated care settings, which may be a barrier for such care.

#### Implications

It seems to be of great importance to better inform caregivers in chronic care about the risk of normalising depression and the magnitude of stigma patients experience about depression. Pro-actively educating patients in chronic care on possible comorbid depression and how to handle such symptoms might further help to diminish experienced stigma and create more patient awareness of depression. This might further facilitate integrated somatic and mental health care, as patients would get more acquainted with the concept of chronic caregivers discussing mental health, which is something patients do not necessarily expect, potentially interfering with the success of care integration [23]. Additionally, exploring in practice which terms and settings individual patients relate most to seem very relevant to improve the acceptance of mental health care. Addressing sleeping problems, for example, might be an easily accepted starting point for patients with (subthreshold) depression. More research on how to best identify mild depressive disorders in patients with DM2/CHD and what prompts patients to accept and seek care could contribute to the success of future depression prevention programs.

#### Strengths and limitations

An important strength of this paper is that both patients and practice nurses were interviewed. Deeper understanding was gained of the caregivers’ and patients’ views, which led to valuable complementary and contrasting data. The utilisation of two analysts, the systematic development of codes and code definitions, the use of a qualitative computer program, and complete data saturation while still conducting interviews, enhanced the quality of the data.

As the organization of care, for example concerning the role of practice nurses in primary care, might be different outside the Netherlands, these findings might be less applicable in other settings. Furthermore, the results are based on the perceptions of patients who participated in the Step-Dep study. It would be of much added value to interview screened patients who did not consent to participate in Step-Dep, as they might have different perceptions of the investigated themes. Additionally, since our interviewees were mainly native Dutch, cultural differences that may influence depression perceptions were not explored.

## Conclusion

Data of the interviewed patients and practice nurses suggest that they have different perceptions about (subthreshold) depressive illness and the need for care, although views on its causes seem to overlap more.

## Appendix 1

**Table 3:** Topic list

RE-AIM	Topic
Reach	Appropriateness Step-Dep patients (target population)
	Depression: recognition, severity, causes, improving factors
	Need for care
	Motivation to participate
	Access mental health care
Efficacy	Perceived effectiveness
	Perceived usefulness
Adoption	Information practices, caregivers
Implementation	Barriers & facilitators
	Deviations from protocol
	Reasons for dropout
	Prerequisites for implementation
Maintenance	Satisfaction
	Feasibility for future

## Appendix 2

**Table 4:** Patients interview

Topic	Question
General	How was your experience participating in Step-Dep/ the program in your general practitioner practice?
	What was the best part for you?
	What was the weakest part for you?
Motivation	Why did you decide to participate in Step-Dep?
Mental state	How would you describe your mental state before starting Step-Dep?
	If not depressed: please tell more about it?
	If depressed: please tell more about it? Did it influence your life? What do you think caused it? Is there a relationship with your chronic disease? How? How is your mental state now? If improved: what are the reasons for that improvement?
	Did you feel the PHQ-9 reflected your mental state correctly? Why? Why not?
Need for care	Were you in need of care/ a preventive program to improve depressive symptoms?
	How would it have been, if you had not received an invitation for Step-Dep?
	What were your expectations/ hopes from the program?
	Did the program match your needs?
	What would your care of choice have been like? And to improve depressive symptoms?
	How would it have been for you to be offered a program at the time of diagnosis of your chronic disease?
Perceived effectiveness	Was the offered program useful to improve your depressive symptoms? Why? Why not? What was most useful to you? How do you see that in the long-term?
	How were/was the consultations with the practice nurse/ self-help/ problem solving treatment/ referral to general practitioner for you?
Suggestions for future care	Would you recommend this program to others? Why? Why not? To whom?
	What would your suggestions be to improve Step-Dep?
	Is there anything you would like to add to the interview?

## Appendix 3

**Table 5:** Practice nurses interview

Topic	Question
General	How did you experience executing Step-Dep?
	What is your opinion on the Step-Dep program?
	What were the main facilitators?
	What were the main barriers?
Reach	Were the selected patients appropriate for this prevention program? Why? Why not?
	How did you view their mental state/ depressive symptoms? Did patients recognize themselves in the depressed profile? What are causes for depressive symptoms? How do you view the relationship with the chronic disease? What coping strategies do patients have with a chronic disease?
	Were the patients in need for care for depression? Other need for care? Why? Why not?
Efficacy	Did the program match their need for care?
	Was Step-Dep effective in your opinion on preventing depression/ improving depressive symptoms for these patients? Why? Why not? How?
	What is your view on the program elements: consultations, self-help, problem solving treatment, referral to general practitioner?
	If the depressive symptoms improved in your patients; what was the reason for this improvement? Did the program play a part?
Implementation	Why did you decide to participate in Step-Dep?
	How do you view your competences to execute the program?
	Was it necessary to deviate from the protocol? Why? Why not?
	How was using the PHQ-9 for you? And as a screening/ monitoring/ decision tool?
	How much time would you need for the consultations/ self-help/ problem solving treatment?
Maintenance	Is this program (or elements) useful in daily practice for this group? Why? Why not?
	Would you use this program (or elements) in the future? Why? Why not?
	What would be necessary to implement this in your practice?
	How would you ideally see depression prevention?
	What is your opinion on offering a program like that at the time of diagnosis of the chronic disease?

## Appendix 4

**Table 6:** Patient and practice nurse characteristics

Patients							
Interview nr	Age	Sex	DM2/ CHD	Educational level	Self-reported depression at baseline*	Self-reported History of depression	PHQ-9 score at inclusion
P1	66	f	CHD	high	no	yes	7
P2	61	f	CHD	high	no	yes	7
P3	63	f	Both	intermediate	yes	yes	9
P4	84	f	CHD	low	yes	no	10
P5	53	f	DM2	high	no	yes	16
P6	72	m	CHD	intermediate	no	yes	10
P7	56	m	DM2	high	no	yes	10
P8	73	f	Both	low	no	no	11
P9	55	m	Both	intermediate	no	yes	14
P10	48	m	DM2	intermediate	yes	yes	12
P11	61	m	DM2	low	yes	yes	8
P12	56	f	Both	high	yes	yes	14
P13	66	m	CHD	high	no	yes	7
P14	57	m	DM2	intermediate	no	no	14
P15	55	f	CHD	low	no	no	15
Practice nurses							
Interview nr		Practice nurse type			Number of Step-Dep patients treated		
N1		psychological practice nurse			24		
N2		psychological practice nurse			15		
N3		psychological practice nurse			13		
N4		psychological practice nurse			10		
N5		somatic practice nurse			3		
N6		somatic practice nurse			6		
N7		psychological practice nurse			15		
N8		currently somatic practice nurse, previously psychological practice nurse			3		
N9		psychological practice nurse			7		

Abbreviations: *F* female, *M* male, *CHD* Coronary Heart Disease, *DM2* Type 2 Diabetes Mellitus, *PHQ-9* Patients Health Questionnaire 9 score. *Scores do not equal inclusion PHQ-9 scores due to time between inclusion and baseline

## Abbreviations

CHD: Coronary heart disease
DM2: Type 2 Diabetes Mellitus
GP: General practitioner
PHQ-9: Patients Health Questionnaire 9
PN: Practice nurse
